# The Role of the Gut Microbiota in the Development and Progression of Major Depressive and Bipolar Disorder

**DOI:** 10.3390/nu14010037

**Published:** 2021-12-23

**Authors:** Tom Knuesel, M. Hasan Mohajeri

**Affiliations:** Department of Anatomy, University of Zurich, Winterthurerstrasse 190, 8057 Zürich, Switzerland; tom.knuesel@uzh.ch

**Keywords:** depression, affective disorder, gut-brain-axis, bacteria, probiotics, therapy, treatment

## Abstract

A growing number of studies in rodents indicate a connection between the intestinal microbiota and the brain, but comprehensive human data is scarce. Here, we systematically reviewed human studies examining the connection between the intestinal microbiota and major depressive and bipolar disorder. In this review we discuss various changes in bacterial abundance, particularly on low taxonomic levels, in terms of a connection with the pathophysiology of major depressive and bipolar disorder, their use as a diagnostic and treatment response parameter, their health-promoting potential, as well as novel adjunctive treatment options. The diversity of the intestinal microbiota is mostly decreased in depressed subjects. A consistent elevation of phylum Actinobacteria, family Bifidobacteriaceae, and genus *Bacteroides*, and a reduction of family Ruminococcaceae, genus *Faecalibacterium*, and genus *Roseburia* was reported. Probiotics containing *Bifidobacterium* and/or *Lactobacillus* spp. seemed to improve depressive symptoms, and novel approaches with different probiotics and synbiotics showed promising results. Comparing twin studies, we report here that already with an elevated risk of developing depression, microbial changes towards a “depression-like” microbiota were found. Overall, these findings highlight the importance of the microbiota and the necessity for a better understanding of its changes contributing to depressive symptoms, potentially leading to new approaches to alleviate depressive symptoms via alterations of the gut microbiota.

## 1. Introduction

Psychiatric disorders belong to the world’s most disabling diseases, particularly major depressive disorder (MDD, unipolar disorder) and bipolar disorder (BD). Approximately 4.4% of the world’s population is affected by depression. According to the World Health Organization, depression is the largest contributor to global disability and “non-fatal health loss”, as well as the major contributor to suicide deaths [1]. Patients with MDD show typical symptoms of sadness, loss of interest and pleasure, feelings of low self-worth, guilt and tiredness, disturbed sleep, and poor concentration. BD is characterized by episodes of depression and mania, separated by episodes of normal mood. Mania includes elevated mood, increased energy and activity, pressure to speech, and decreased need to sleep [1].

In twin and family studies, heritability rates, defined as genetic factors contributing to the occurrence of a certain disease, were found to be moderate in MDD [2], and high in BD [3,4]. Despite significant advances, the pathogenesis of MDD and BD is still not fully understood. The diagnosis is based only on clinical symptoms, and a high rate of treatment resistance is observed [5]. Poverty, unemployment, severe life events, physical illness, and the consumption of alcohol and drugs are risk factors, but anyone can be affected by depression [1]. Especially functional gastrointestinal disorders (FGID) like irritable bowel syndrome (IBS) are often associated with depression, with the co-occurrence estimated at 30% [6]. Altered neurotransmission, changes in the hypothalamic-pituitary-adrenal axis (HPA axis), chronic low-grade inflammation, reduced neuroplasticity, and neuronal network dysfunction probably contribute to the pathogenesis of depression [7]. IBS pathogenesis shares several of these changes, indicating a multifactorial association between both diseases [8]. Additional evidence suggests a connection between depression, increased gut wall permeability, and bacterial translocation, resulting in increased immune activation and inflammation, with the intestinal microbiota being an important contributor [9,10].

The human gut microbiota consists of an estimated number of 3.8 × 10^13^ (38 trillion) bacteria, containing slightly more bacteria than cells of the human body (approximately 3.0 × 10^13^), and by far more genes than its human host [11]. In addition, after the brain, the human gut contains the second greatest number of neurons. Heritability rates of the gut microbiota in humans were estimated between 1.9% and 8.1% [12]. A disturbed intestinal microbiota, often associated with reduced diversity, was found in a variety of diseases, including hypertension [13], obesity [14], gastrointestinal disorders (such as inflammatory bowel disease (IBD) [15,16], and IBS [17]), brain disorders (such as Alzheimer’s disease [18], Parkinson’s disease [19], autism spectrum disorder [20], and attention-deficit/hyperactivity disorder [21]), autoimmune diseases [22], as well as some types of cancer (for example colorectal cancer [23]). It was even suggested that a disturbed intestinal microbiota in obese patients may be a further reason for increased coronavirus disease 2019 (COVID-19) severity [24].

The intestinal microbiota can interfere with the HPA axis. Stress-induced stimulation of the HPA axis leads to elevated adrenocorticotrophin (ACTH) release and therefore results in a higher glucocorticoid excretion. In restraint-stressed germ-free mice, elevated ACTH and corticosterone (a glucocorticoid) levels were found, compared to specific pathogen-free mice [25], showing a direct connection between the HPA axis and the microbiota. The HPA axis can also be influenced through metabolites produced by the intestinal bacteria, like cytokines and prostaglandins, leading to exaggerated or attenuated stress response [26]. The gut microbiota can break down otherwise indigestible food substances and produce micronutrients [27], short-chain fatty acids (SCFAs) [28], neurotransmitters such as gamma-aminobutyric acid (GABA) [29], and brain active non-SCFA metabolites [18]. Acetate, propionate, and butyrate are the most abundant SCFAs in the human intestine [30]. They can influence emotion, cognition, and the immune system. In particular, a correlation between higher depression scores and lower levels of acetate and propionate was found in women, while sodium butyrate reversed depressive and manic symptoms in mice and was suggested as a mood stabilizer in humans [31,32]. Bacterial metabolites can translocate out of the gut and interact with the HPA axis, with the immune system, and with vagal afferents, leading to an exaggerated (or attenuated) HPA response and consequently to a modulation of the immune system [26,33,34]. Further research reported a systemic chronic low-grade inflammation in mice models, as well as in a significant proportion of depressed subjects, suggesting the presence of a mucosal dysfunction in depressed individuals, leading to an elevated translocation of intestinal bacteria into the circulation [35,36,37]. Consequently, an increased antibody response against lipopolysaccharides (LPS) from gram-negative bacteria is induced in diseased individuals [9]. In mice, intraperitoneally injected LPS caused a depressive-like behavior, and a following treatment with sodium butyrate ameliorated these changes, underlining the negative influence of translocated bacteria and LPS, as well as the positive influence of butyrate on the depression pathophysiology [35].

Several possible connections between the intestinal microbiota and depression are currently being discussed. The gut microbiota is considered to be under-explored, and its detailed investigation is needed for revealing specific associations. Most studies examining a possible connection between the gut microbiota and depression are conducted in rodents, while human research is still lagging. Hence, we systematically reviewed the connection between the human intestinal microbiota and major depressive and bipolar disorder, intending to analyze which bacteria could possibly influence depression or vice versa, and which bacteria future studies should primarily focus on.

## 2. Materials and Methods

The main question of this review was whether there is a connection between the intestinal microbiota and major depressive and bipolar disorder in human subjects. Does the intestinal microbiota influence the development, severity, and remission of affective disorder? The databases Scopus and PubMed were searched until 1 May 2020, with the following MeSH and search terms: “microbiota”, “microbiome”, “depression”, “depressive”, “bipolar disorder”.

Additional inclusion criteria were as follows:

- Studies were written in English
- Studies were conducted with human subjects
- Studies at least partly focused on depression or depressive symptoms, and their correlation with the intestinal microbiota
- Diseased and healthy subjects were analyzed in the study

We focused on bacterial taxa and therefore excluded results regarding fungi, archaea, and viruses. Studies investigating microbiomes other than the intestinal microbiota were also excluded. We included all studies related to MDD, BD, and the intestinal microbiota, leading to the high heterogeneity of the reports, but providing a comprehensive overview of published data on this topic.

Twelve articles were excluded after full-text assessment due to not focusing on depression, depressive symptoms (*n* = 9) or the intestinal microbiota (*n* = 3).

**Figure 1:**
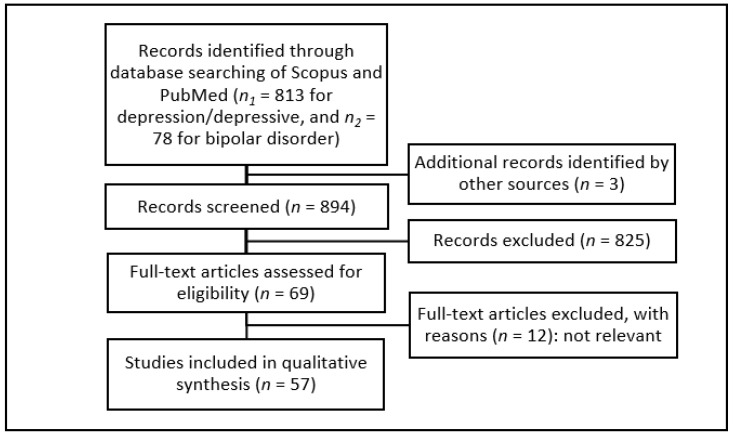
Methodical approach of our review due to PRISMA criteria [38].

A total of 57 studies were included in this review (Figure 1), most of which were published between 2016 and 2020, demonstrating a rapidly growing interest in this topic in recent years.

## 3. Results

### 3.1. Diversity

Microbial diversity can be specified as alpha-diversity and beta-diversity. Alpha-diversity describes the species richness and evenness (inequality of the relative abundance) within a sample. In the reviewed studies it was most often determined by using the Shannon index, but several measures are common for richness and evenness estimation, as the ACE-, Chao1-, and Simpson index, phylogenetic diversity, and the number of observed species [39]. Beta-diversity describes the difference between multiple samples and is mostly analyzed by using unweighted/weighted UniFrac distances and Bray-Curtis dissimilarity [40]. Additionally, PLS-DA (partial least squares discriminant analysis) was used to detect microbial patterns that separate depressed subjects from healthy controls (HC) [41].

Apart from Jiang et al. [42] who reported an increased alpha-diversity in active-MDD subjects, all alpha-diversity indices, and other measures were consistently reported to be equal or reduced in depressed subjects among the other studies. For example, four studies reported a negative correlation between the Shannon index and depression, while 13 reported no correlation. Similar results were found concerning the other above-mentioned indices and measures for “within-sample” alpha-diversity (Table 1).

Regarding “between-samples” beta-diversity changes, a correlation with depression could be found in most studies. Both studies comparing MDD and BD subjects to HC using Bray-Curtis dissimilarity found a significant difference [43,44]. Regarding unweighted and weighted UniFrac distances, studies reported contradictory results, and no final statement can be made as to whether depressed subjects show different UniFrac distances compared to HC (Table 1).

Using PLS-DA, all four studies found significant differences between the depressed and the HC group. In addition, with a PLS-DA model, Li et al. found significant correlations between microbial and mood changes in healthy adults over time [45].

**Table 1:** A selection of the most used diversity indices and measures, showing an unchanged or lower microbial diversity in depressed individuals.

Source	Shannon	Ace	Chao1	Nr. OTUs	UniFrac	PLS-DA
[41]				D =		D sign.
[42]	aMDD ↑, rMDD =	MDD =	MDD =		MDD =	
[43]	MDD =					
[44]	MDD =				MDD =	
[46]		BD ↓, MDD =	BD ↓, MDD =			BD, MDD, C sign.
[47]	P =				P =	
[48]	P ↓			P ↓		
[49]		MDD =	MDD =			MDD sign.
[50]	MDD =, BD =		MDD ↓, BD =			
[51]	MDD =		MDD =	MDD =	MDD sign.	
[52]	BD =		BD =	BD =	BD =	
[53]	D =			D ↓	D =	
[54]		BD ↓ *	BD ↓ *		BD sign.	
[55]			D = IBS	D = IBS	D, IBS sign.	
[56]	IBS ↓				IBS sign.	
[57]	MDD =			MDD =		MDD sign.
[58]	MDD ↓	MDD ↓	MDD ↓		MDD sign.	
[59]	pm =					
[60]	D ↓, IBS ↓					
[61]	D ↓			D ↓		
[62]					MDD sign.	
[63]	MDD =				MDD sign.	
[64]	BD =			BD =	BD sign.	
[65]	BD =			BD sign.		

“UniFrac” includes weighted and/or unweighted UniFrac distances. “↓” shows a significantly reduced diversity in diseased subjects compared to controls or an inverse correlation with more severe symptoms, while “↑” indicates a significantly increased diversity or a positive correlation with more severe symptoms. “=” demonstrates no significant difference, while “sign.” shows a significant difference. Empty cells symbolize that no results were reported. Abbreviations: MDD, major depressive disorder; BD, bipolar disorder; C, control group; P, psychiatric subjects; D, depression in general; IBS, irritable bowel syndrome; a, active disease group; r, response group; pm, psychiatric measures; OTU, operational taxonomic unit; *, only showing a trend, due to small sample size.

Overall, the microbial diversity of depressed individuals was reported unchanged or reduced, compared to the general population. These findings support the hypothesis that the intestinal microbiota is connected with the development, preservation, and remission of depression. With a better understanding of this link, depressive symptoms could potentially be positively influenced by specific alterations of the microbiota. Our analysis also shows that a reduced diversity is not present in all depressed subjects, and therefore cannot be used as a reliable diagnosis parameter. However, using the diversity change over time as a treatment response and prognosis parameter could be possible, but whether this is clinically feasible remains to be proven.

### 3.2. Phylum

On the phylum level, Bacteroidetes phylum was reported to be reduced in depressed subjects in multiple studies, but other studies reported opposite results (Table 2). Important to mention is that Chen et al. reported an elevation of Bacteroidetes in young MDD individuals, while in middle-aged MDD individuals Bacteroidetes were reduced, compared to age-matched controls [49]. On the one hand, comparing the studies which reported an elevation of Bacteroidetes in depressed patients, Liu et al. [44] only included MDD subjects between age 18 and 25, Hu et al. [66] used a young BD group (mean age 24 years), with an older, not age-matched control group, and Jiang et al. [42] only included MDD patients aged 40 or younger. On the other hand, studies reporting a reduction of Bacteroidetes correlating with depression were mostly conducted with older subjects. For example, in the study by Lai et al. [43], MDD individuals were between 32 and 52 years old, in Rong et al. [50] the mean age of all groups was between 38 and 42 years, and in Chen et al. [67] MDD subjects were 44 years old on average. Summarized, strong evidence was found that in young patients suffering from affective disorder, phylum Bacteroidetes is elevated, while in middle-aged patients these bacteria are reduced, compared to age-matched HC. This could point towards different causes of depression or a different manifestation of depressive behavior with age, leading to a different microbiota in depressed subjects.

The phylum Firmicutes was reported to alter in both directions (Table 2). However, most studies reporting an increase of Firmicutes also found a reduction of Bacteroidetes, or vice versa. Above mentioned changes due to different age ranges can also be applied to the phylum Firmicutes. Chen et al. [49] found lower Firmicutes mainly in the young MDD group, and Jiang et al. [42], Liu et al. [44], and Hu et al. [66] all studied young MDD or BD subjects and reported a reduced abundance of Firmicutes in the depressed group. Studies with older (middle-aged) subjects rather reported an elevation of Firmicutes abundance (Table 2).

Concerning the phylum Proteobacteria, results were highly controversial. Zheng et al. [46] and Rong et al. [50] both reported an elevation in BD, but not in MDD subjects, suggesting a potential difference between BD and MDD. In contrast, Hu et al. [66] found reduced Proteobacteria in untreated, compared to treated BD subjects, but not compared to HC. A recent review reported a lower abundance of phylum Proteobacteria in healthy subjects, while an elevated abundance was associated with a variety of diseases, including IBD, metabolic disorder, or malnutrition [68]. However, a link between phylum Proteobacteria and depression could not be shown, and neither age-dependent changes nor a difference between BD and MDD subjects could be further supported in this review.

The phylum Actinobacteria was consistently found to be elevated in MDD or BD individuals. Nine studies reported an elevation, while none found a decrease of Actinobacteria in patients with affective disorder (Table 2). Therefore, strong evidence for a close connection between an elevation of phylum Actinobacteria and depression was found, and more attention should be paid towards this phylum, looking for possible causes and consequences of an increase in Actinobacteria abundance.

**Table 2:** Different abundance on phylum level, with the studies sorted by age of the diseased subjects.

Source	Mean Age (Years)	Bacteroidetes		Firmicutes	Proteobacteria	Actinobacteria
[44]	MDD 21.9; C 22.1	MDD ↑		MDD ↓		
[49]	MDD 24.0; C 25.0	MDD ↑		MDD ↓		
[66]	BD 24.2; C 36.3	BD ↑		BD ↓	BD ↓ *	
[42]	aMDD 25.3; rMDD 27.1; C 26.8	MDD ↑		MDD ↓	MDD ↑	MDD ↑
[46]	MDD 26.5; BD 25.6; C 26.9	MDD ↑	BD ↓		BD ↑	
[69]	non-D 33.4				↓	
[48]	P 35.7			↓		
[62]	MDD 36.2; C 38.1	MDD ↓		M ↑		MDD ↑ *
[63]	MDD 40.6; C 41.8	MDD ↓				MDD ↑
[52]	BD 41.3; C 31.4					BD ↑
[57]	MDD 41.5; C 44.0	MDD ↓				MDD ↑
[50]	MDD 41.6; BD 38.4; C 39.5	MDD ↓; BD ↓		MDD ↑; BD ↑	BD ↑	MDD ↑; BD ↑
[43]	MDD 43.7, C 39.4	MDD ↓				MDD ↑
[67]	MDD 43.9; C 39.6	MDD ↓		M ↑	MDD ↓	MDD ↑
[60]	D 44.8; IBS 38.5; D + IBS 39.0; C 43.9	D ↑		D ↓		
[55]	IBS + Di 45.0; IBS (non-Di) 33.0				↑	
[49]	MDD 45.0; C 47.2	MDD ↓				MDD ↑
[51]	MDD 45.8; C 41.2	MDD ↓		MDD ↑	MDD ↓	MDD ↑
[58]	MDD 48.7; C 42.3			MDD ↓		
[41]	D 49.2; C 46.1	D ↓				

Elevated Bacteroidetes and reduced Firmicutes were found in young-aged, depressed subjects, while in middle-aged those phyla showed opposite correlations. Actinobacteria was consistently increased. “↓” and green symbolizes a significant reduction in diseased subjects or inverse correlation with more severe symptoms, while “↑” and orange shows a significant elevation or positive correlation with more severe symptoms. Grey symbolizes only a trend, while empty cells symbolize that no significant results were reported. Abbreviations: MDD, major depressive disorder; BD, bipolar disorder; C, control group; D, depression in general; non-D, non-depressed subjects; Di, distressed subjects; IBS, irritable bowel syndrome; P, psychiatric subjects; a, active disorder group; r, response group; *, only showing an insignificant trend.

Summarized, age seemed to firmly influence bacterial abundance. While in young-aged patients Bacteroidetes were elevated and Firmicutes reduced, in middle-aged subjects a reduction of Bacteroidetes and an elevation of Firmicutes was reported, compared to age-matched HC, whereas Actinobacteria was consistently elevated regardless of age. However, on lower taxonomic levels correlations could show opposite directions even for closely related bacteria, suggesting that the abundance of a specific phylum is not as decisive as the abundance of certain bacteria on lower taxonomic levels, i.e., on the genus or species level.

### 3.3. Bacteroidetes

The phylum Bacteroidetes is the most dominant in the human gut [70]. It contains four important families, namely Bacteroidaceae, Tannerellaceae, Prevotellaceae, and Rikenellaceae.

Most studies reported a correlation between depressive symptoms and the family Bacteroidaceae or genus *Bacteroides*, the most abundant family and genus of the intestinal microbiota [70]. Especially genus *Bacteroides* was repeatedly associated with affective disorder, high anhedonia, and negative mood (Table 3). In general, *Bacteroides* were found to be negatively associated with inflammation [50,71], to contribute to the gut colonization resistance (the resistance against colonization of enteric pathogens), and to produce SCFAs, mostly acetate and propionate, which are important for gut homeostasis [72,73]. *Bacteroides* are known as starch degraders and they potentially cross-feed other species, like *Eubacterium ramulus*, which in turn can produce beneficial molecules like butyrate, and therefore reduce gut hyperpermeability by increased expression of tight-junctions [72]. Even though genus *Bacteroides* was found to provide beneficial effects on the human host, the family Bacteroidaceae and genus *Bacteroides* were repeatedly found to be elevated in depressed subjects. While some of the reviewed studies were conducted only with few individuals, Cheng et al. included thousands of subjects, strongly supporting this correlation [74]. These findings may hint at a compensatory mechanism and suggest that the alteration in the abundance of a certain bacteria may not necessarily have negative health effects. A higher taxonomic resolution could lead to more precise information about these correlations.

Genus *Parabacteroides* of the Tannerellaceae family tended to be elevated in depressed subjects, but two studies reported contrary results [60,69]. These two studies used a small sample size of depressed subjects (*n* = 15), and reported a reduction in non-depressed participants to correlate with anxiety and DASS-42 (depression anxiety and stress scales) scores, but not directly with depression, respectively [60,69]. Investigating the three studies which compared MDD or BD subjects with HC, all three reported an elevation of *Parabacteroides* correlating with depression [42,51,66]. *Parabacteroides* produce SCFAs, especially acetate, and can reduce neutrophils in the blood [76]. Even though they have health-promoting effects, they tended to be more abundant in individuals with affective disorders. Therefore, as with the family Bacteroidaceae, an elevation of *Parabacteroides* could be a compensatory mechanism, rather than unfavorably influencing the host’s mood.

The abundance of the Prevotellaceae family altered in both directions in depressed subjects, with no tendency overall. Worth mentioning is that Chen et al. found a reduction of Prevotellaceae in middle-aged MDD, compared to young-aged MDD individuals [49]. This goes in line with our general findings, with two studies reporting a reduced Prevotellaceae abundance in middle-aged MDD subjects (mean age 45.8 and 43.9 years, respectively) [51,67]. On genus level, *Prevotella* inversely correlated with depression, lower mood, or lower quality of life in four studies [42,45,51,77], while others reported a positive correlation [60,62]. Therefore, the suggestion of Lin et al. [62], to use changes of *Prevotella* and *Klebsiella* for laboratory diagnosis and treatment evaluation in MDD, could not be further supported regarding *Prevotella* changes, because the results showed no clear tendency and additional studies even found an opposite correlation. Concerning *Klebsiella* changes, more research is needed to be able to draw a conclusion (further information in the paragraph “Proteobacteria”). Interestingly, two studies conducted with IBS subjects reported an elevation of Prevotellaceae, as well as an elevation of *Prevotella* and *Paraprevotella* to correlate with depressive symptoms, indicating a potential correlation with IBS and comorbid depression [55,60]. However, due to small sample sizes and different study designs of these two studies, additional research focusing on bacterial alterations in IBS subjects is required.

Within the family Rikenellaceae, results showed an elevation of genus *Alistipes* or operational taxonomic units (OTUs) within this genus to correlate with depression [41,42,63]. While *Alistipes* seemed to attenuate the severity of colitis via attenuating the expression of anti-inflammatory cytokines in mice, this genus was found to be increased in stressed mice, as well as in patients suffering from chronic fatigue syndrome. It is proposed to decrease serotonin concentration and therefore negatively influence the gut-brain axis, which is in line with our conclusion that an elevated *Alistipes* abundance is associated with unfavorable health effects and potentially promotes the pathogenesis of depression [78]. More studies are needed to further investigate the influence of the Rikenellaceae family on depression.

### 3.4. Firmicutes

The phylum Firmicutes is the second most abundant phylum in the human intestinal microbiome [70]. It was also the most changed, as well as the most discussed phylum within the reviewed studies.

Class Bacilli includes two important families, the families of Lactobacillaceae and Streptococcaceae. *Lactobacillus* bacteria are widely known for their beneficial health effects and their use as probiotics. Despite several studies reporting a higher abundance of *Lactobacillus* to be associated with diverse positive factors like sleep and self-judgment, no direct correlation of *Lactobacillus* abundance and affective disorder could be identified [79,80]. Family Streptococcaceae and genus *Streptococcus* showed a positive correlation with depression and lower quality of life scores. For example, beta-hemolytic Streptococcus Group A infections are known to potentially cause Pediatric Autoimmune Neuropsychiatric Disorders (Associated with Streptococcal Infections, “PANDAS”), which are associated with alterations in the gut microbiome and the nervous system [81]. Although not fully understood, it demonstrates that *Streptococcus* infections can lead to an autoimmune response, severe brain alterations, disturbed neurotransmitters, and can cause psychiatric symptoms like obsessive-compulsive disorder, tics, anxiety, and sometimes even depression [82]. Of the included studies, four reported an elevation of Streptococcaceae (or OTUs within this family) and *Streptococcus* in MDD and BD [50,51,62,63]. Additionally, investigating a large cohort, Valles-Colomer et al. found a negative association between *Streptococcus* and body pain, but no direct association with depression, maybe due to the cohort representing the general population and not being limited to specifically MDD or BD subjects [77]. Hence, there is a need for more studies on the Streptococcaceae family and *Streptococcus* genus regarding their influence on mental health, with potential for novel therapeutic approaches.

Concerning the class Clostridia, a reduction of class Clostridia or order Clostridiales seemed to correlate with worse health and depressive symptoms. However, on lower taxonomic levels, probably due to the diversity of the family Clostridiaceae [83] and an insufficient number of well-controlled studies, no clear association could be identified between Clostridiaceae or *Clostridium* and depression, and studies reported ambiguous findings. There is a need for in-depth studies at high taxonomic resolution to further investigate a potential connection between certain genera or species within the family Clostridiaceae and affective disorder. In addition, an antidepressant therapy with adjunctive probiotic *Clostridium butyricum MIYAIRI 588* showed a high response rate with significant improvement of depressive symptoms in treatment-resistant MDD subjects (further information in the paragraph “human interventional trials in depression”) [84]. Family Christensenellaceae and Christensenellaceae R-7 group were reported to be less abundant in subjects with affective disorder and to inversely correlate with more severe symptoms and higher anhedonia in three studies, while none reported opposite results [44,47,53]. Family Christensenellaceae has been shown to produce acetate and butyrate, and was negatively associated with visceral fat mass [85,86]. Even though, to our knowledge, not much is known about this family so far, a higher abundance of family Christensenellaceae is related to beneficial health effects, while depression correlates with a reduction of this family.

Family Peptostreptococcaceae or genus *Peptostreptococcus* were associated with depression [51,58] and anxiety [69] in three studies. In general, while some studies found a connection between *Peptostreptococcus* species and colorectal cancer [87], others proposed a beneficial effect via the production of indoleacrylic acid (a metabolite of tryptophan), improving the intestinal epithelial barrier function, as well as suppressing inflammatory response [88]. Despite these potentially beneficial effects, a rather negative correlation between *Peptostreptococcus* and depression was reported in the reviewed studies. But with only two studies finding a significantly altered abundance in small cohorts of depressed individuals, while none of the studies with more subjects reported similar results, an important association with depression is unlikely.

Family Eubacteriaceae and especially species *E. rectale* tended to be more abundant in healthy subjects and increased after antipsychotic BD treatment with quetiapine [44,47,57,75]. *Eubacterium* spp. can produce propionate and butyrate, and therefore suppress inflammation, enhance the intestinal barrier integrity, and thereby benefit the host’s health [89]. This is in line with our findings, where Eubacteriaceae correlated with better general health. But the evidence in terms of an association with depression remains scarce.

Family Lachnospiraceae is the second most abundant family in the human gut [70]. On the family level, studies did not show a clear connection between Lachnospiraceae and depression, with Lachnospiraceae being altered in both directions in depressed individuals. However, on the genus level, several inverse correlations with depression were found. Genus *Coprococcus* and OTUs within this genus were found to be less abundant in depressed subjects and to correlate with higher quality of life [46,48,58,60,63,66,77]. One study found specifically *C. catus* to be less abundant in subjects with more severe depressive symptoms and to positively correlate with remission [48]. Genus *Coprococcus* is known for its butyrate production [90], and previous research found a reduced *Coprococcus* abundance in several diseases, like inflammatory bowel disease [16], colorectal cancer [91], and preeclampsia [90]. According to Zhang et al., *Coprococcus* abundance can be increased by omega-3 polyunsaturated fatty acids (PUFAs), while lower levels of omega-3 PUFAs were found in depressed subjects [92,93]. Therefore, a connection between genus *Coprococcus*, PUFAs (especially omega-3 PUFAs), and depression is imaginable, emphasizing the importance of a healthy diet and its influence on the intestinal microbiota and depression. The abundance of genus *Fusicatenibacter* or unclassified species within this genus were reduced in depressed subjects and associated with a higher quality of life in three studies, indicating a slightly beneficial effect of these bacteria [44,55,77]. Due to contradictory results concerning genus *Blautia*, no final statement could be made regarding a link of genus *Blautia* with depression. Genus *Roseburia* or OTUs within this genus were mostly reported to be reduced in subjects with depressive symptoms and correlated with remission and positive mood (Table 4). Research showed that *R. intestinalis* suppressed inflammation and promoted anti-inflammatory cytokines in a colitis mouse model, and found *Roseburia*, together with *Faecalibacterium*, to be one of the most abundant known butyrate-producing bacteria in the human gut [94,95]. This is in line with our findings of *Roseburia* being reduced in depressed individuals. Therefore, an increase of bacteria belonging to the genera *Roseburia* or *Coprococcus* may provide beneficial physical and mental health effects, and further investigation is needed as to whether this effect can be used in the treatment of affective disorder.

Concerning the family Ruminococcaceae, most studies found a higher abundance in healthier subjects (Table 5). Interestingly, the abundance of family Ruminococcaceae was associated with remission, but several taxa within Ruminococcaceae positively correlated with symptom severity of psychiatric subjects [48]. Additionally, both increased and decreased OTUs within this family were found in MDD patients, underlining the differences of bacterial abundance on low taxonomic levels [49,57]. On genus and species level, on the one hand, multiple studies reported a higher abundance of genus *Oscillibacter* in depressed subjects [41,42,43,50]. Genus *Oscillibacter* is suggested to be elevated rather as a result of depression, due to its potential to metabolize proteins [96]. Underlining this, in MDD subjects, disturbed bacterial proteins were found, which are involved especially in metabolic pathways related to amino acid metabolism [67]. On the other hand, genus *Ruminococcus*, *Gemmiger*, and especially *Faecalibacterium* were more abundant in healthier subjects in the majority of the reviewed studies (Table 5). *Faecalibacterium* is known for its butyrate production, anti-inflammatory potential, and intestinal barrier function improvement, and was suggested as a probiotic for IBD, gut dysfunction, and low-grade inflammation treatment [95,97,98]. In the reviewed studies, a negative correlation with depressive symptoms and a positive correlation with remission and higher quality of life was reported, highlighting the potential of probiotic *Faecalibacterium* as a novel treatment option, and their abundance as a parameter for diagnosis or treatment response. Even though several studies reported opposite correlations, contradictory results can mostly be explained by very small sample sizes, age and sex differences, and by not unexceptionally used false discovery rate. In conclusion, the family Ruminococcaceae is a perfect example that even closely related bacteria can show an altered abundance in opposite directions. Even though on the family level, most of the studies reported a higher abundance in healthier subjects, on lower taxonomic levels bacterial alterations were found in both directions. Similar to other families, a higher taxonomic resolution of this family and its genera is needed for a more specific examination of these bacteria and their interaction with the host, with especially genus *Faecalibacterium* showing a close negative association with depression.

Additionally, an elevation of genus *Flavonifractor* was associated with depression, symptom severity, or worse physical functioning, with no contradictory results [42,44,48,64,77]. Even though *Flavonifractor plautii* was recently found to suppress the immune response in mice in multiple studies conducted by the same research group [101,102,103], it was repeatedly associated with several diseases, including ulcerative colitis, autoimmune diseases, obesity, and even with a poor diet [104,105]. Our findings of elevated *Flavonifractor*, with no study finding opposite results, strongly support its negative influence on the host’s health, including affective disorder.

### 3.5. Proteobacteria

Within the phylum Proteobacteria, most differences between depressed individuals and HC were found within order Burkholderiales of class Betaproteobacteria. Five studies reported a reduction of family Sutterellaceae, OTUs within this family, or genus *Sutterella* in depressed subjects [51,57,60,63,67]. Additionally, Peter et al. [55] reported an association between order Burkholderiales abundance and perceived stress, which is inconsistent with the other results on lower taxonomic levels, demonstrating that a high taxonomic resolution should be striven for. No study found elevated Sutterellaceae or *Sutterella* in patients with affective disorder. Hence, a negative association with depression is conceivable on these taxonomic levels. Even though not very much is known about this family, it seems to be associated with diseases like autism spectrum disorder, down syndrome, and IBD. Furthermore, a mild pro-inflammatory capacity of certain species within genus *Sutterella* was proposed [106]. Therefore, the origin and consequence of reduced Sutterellaceae and *Sutterella* in depressed individuals remain unclear.

Within class Gammaproteobacteria, family Enterobacteriaceae tended to be elevated in subjects with affective disorder, but with controversial results. At the genus level, few studies reported a higher abundance of *Enterobacter* and *Klebsiella* to correlate with worse health [54,62,75]. The family Enterobacteriaceae, with its well-known genera *Enterobacter*, *Escherichia*, *Klebsiella*, *Salmonella*, and *Shigella*, is associated with many different clinical syndromes and diseases, including foodborne infectious diarrhea, enteritis, colitis, hemolytic uremic syndrome, as well as extraintestinal diseases [107]. Maes et al. found increased serum immune globulin M (IgM) against LPS of Gammaproteobacteria in depressed individuals, highlighting the link between intestinal mucosal dysfunction, increased bacterial translocation, immune response, and depression [9]. Family Pseudomonadaceae and genus *Pseudomonas* were elevated in depressed subjects in two studies, but with controversial results regarding only MDD subjects [46,58]. Maes et al. also found increased IgM against *Pseudomonas* in MDD subjects, compared to HC [33]. Here too, as concluded for other families, more detailed information on lower taxonomic levels is needed for a clear-cut statement.

Genus *Desulfovibrio* of class Deltaproteobacteria seemed to positively correlate with MDD and BD, but only three of all reviewed studies found a different abundance [44,57,74]. Cheng et al. analyzed published genome-wide association study data sets with high numbers of cases and controls [74]. They reported an association of genus *Desulfovibrio* with MDD, BD, and other mental disorders, suggesting a crucial role of this genus in mental disorders. However, these findings are not consistent with the results of the other two studies, which found *Desulfovibrio* to be elevated only in female but not in male MDD subjects, and even reported an inverse correlation with MDD, respectively [44,57]. Age could be an important confounding factor, due to young participants in Liu et al. [44] and middle-aged in Chen et al. [57]. Therefore, genus *Desulfovibrio* could be reduced in young-aged, depressed subjects, while in middle-aged these bacteria could be elevated, but further investigation is needed.

### 3.6. Actinobacteria

Within the phylum Actinobacteria, study results tended towards an increase of class Coriobacteria, order Coriobacteriales, family Coriobacteriaceae, or OTUs within this family correlating with depression, but with inconsistent results [47,49,52,57,63]. However, on the genus level, a higher abundance of genus *Collinsella* was associated with lower anhedonia, BD treatment, and remission [47,48,66]. Only one study reported an association of elevated *Collinsella* with depression scores, suggesting that there is little evidence for a positive correlation with depression [57]. Other research found a stress-induced increase of an unspecified genus of Coriobacteriaceae in mice, a reduction of genus *Collinsella* after weight loss in obese type 2 diabetics, and a positive correlation of *Collinsella* with circulating insulin levels and low dietary fiber intake, while a high fiber intake supports SCFA-promoting gut bacteria [108,109,110]. Therefore, *Collinsella* is generally associated with worse health, and consequently, it remains unclear why especially genus *Collinsella* tended to be associated with ameliorated depressive symptoms. Even though closely related, genus *Eggerthella* was shown to be associated with MDD and higher depression and perceived stress scores in the reviewed research [43,51,57]. An elevated *Eggerthella* abundance was also found in immune-mediated inflammatory diseases like Crohn’s disease and ulcerative colitis [111]. In conclusion, while on a higher taxonomic level an increase of these bacteria was found in depressed subjects, on lower taxonomic levels this consistent increase could not be seen, due to a reduction of genus *Collinsella* correlating with depression.

Within the order Bifidobacteriales, genus *Bifidobacterium* is known for its beneficial effects on the host’s health and a lower abundance is associated with several diseases [112]. Counterintuitively, most studies found a positive association with depression and negative mood, while only three studies reported an elevation correlating with better health or depression treatment (Table 6). While in depressed subjects *Bifidobacterium* abundance seemed to be elevated, most studies reported a significant improvement in depressive symptoms with probiotics containing *Bifidobacterium* spp. (Table 7). The reason for those seemingly contradicting results remains unclear and needs further research.

In general, most of the investigated bacteria belonging to phylum Actinobacteria tended to correlate with worse health and depression, which is again contrary to the general finding of Actinobacteria having a positive influence on human health and its beneficial effects as probiotics [112]. Microbial bacteria are firmly influenced by diet [112], but only few studies included dietary data. Therefore, different eating habits could be a possible factor leading to elevated Actinobacteria in depressed individuals, but a satisfactory explanation is not possible to date.

### 3.7. Human Interventional Trials in Depression

A total 13 studies investigated the influence of probiotic *Lactobacillus* and/or *Bifidobacterium* on depression. While six studies found no significant improvement in depressive scores, seven reported a significant amelioration of depression (Table 7). Three of them were conducted by the same research group, focusing specifically on the species *Lactobacillus gasseri* [114,115,116]. These three studies reported the most positive and most diverse results. According to them, *L. gasseri* ameliorated depression and anxiety, shortened sleep latency and awake time, lightened fatigue, improved global sleep quality, but also lowered salivary cortisol levels, and even suppressed unfavorable intestinal bacteria. However, to our knowledge, no other studies investigated the effect of *L. gasseri* supplementation on depressive symptoms to date. It might be essential to verify these highly encouraging results with *L. gasseri* probiotics by additional independent research groups. Supporting these beneficial findings of *Lactobacillus* and *Bifidobacterium* probiotics, Heym et al. reported a strong correlation between *Lactobacillus* spp. abundance and positive self-judgment, but only an indirect relationship between *Lactobacillus* spp. and depression, while *Bifidobacterium* spp. showed no association with any psychometric measures [80]. However, all but one of the compared studies reported a beneficial effect of these probiotics, and despite often not reaching significance level, depression scores mostly showed a slight reduction. This indicates that probiotic *Lactobacillus* and *Bifidobacterium* have a modest beneficial effect on depressive symptoms. Whether the effect size is large enough to be of clinical importance needs further investigation, but with none of these 13 studies reporting a worsening of depression or other serious side effects in the probiotic groups, probiotic *Lactobacillus* and *Bifidobacterium* should be considered as an adjunctive treatment in the therapy of affective disorder and depressive symptoms.

Additional studies investigated the use of prebiotics, synbiotics, and different probiotics on depression (Table 7). While in the probiotic group depressive scores only tended to ameliorate, in the synbiotic group a significant difference was found by Haghighat et al. [117]. Therefore, an additional supplementation containing fructo-oligosaccharides, galacto-oligosaccharides, and inulin could further support the beneficial effects of probiotic *Lactobacillus* and *Bifidobacterium*. In treatment-resistant MDD subjects, probiotic *Clostridium butyricum MIYAIRI 588*, a butyrate, acetate, and propionate producing bacteria, showed in combination with antidepressant medication not only an improvement of depressive symptoms but also a response rate as high as 70%, with 35% reaching complete remission [84]. These results are very promising and as mentioned before, further studies may pave the way for the use of probiotic *C. butyricum MIYAIRI 588* in depressive patients. The use of no probiotic food or supplementation was associated with higher odds of depression in a large cross-sectional study. However, individuals consuming probiotics were wealthier and showed a healthier lifestyle on average, resulting in a lower risk of developing depression [118]. These data indicate only an indirect link of probiotics and depression and emphasize that not all probiotic bacteria result in lower rates of depression.

**Table 7:** Effect of prebiotics, probiotics, and synbiotics on depression.

Source	Subjects; Pre-/Syn-/Probiotics	Influence on Depression/Depressive Symptoms
[84]	MDD; *C. butyricum*	+(treatment response, remission)
[99]	D; *Lactobacilli*, *Bifidobacteria*	=(BDI, BAI, DASS)
[114]	H; *L. gasseri*	+(depression, anxiety, sleep)
[115]	H; *L. gasseri*	+(HADS, fatigue, mental state)
[116]	H; *L. gasseri*	+(depressive mood, anxiety, sleep, stress)
[118]	C; any probiotics/supplementation	=(PHQ-9)
[117]	Dialysis; *L. acidophilus*, *Bifidobacteria*; fiber	+with synbiotics (HADS, BDNF)=with probiotics (HADS, BDNF)
[119]	MDD; galacto-oligosaccaride; *L. helveticus*, *B. longum*	=with prebiotics (BDI)+with probiotics (BDI)
[120]	MDD; *L. plantarum*	=(HDRS, PSS)+(attention, perceptivity, verbal learning)
[121]	D; *L. helveticus*, *B. longum*	=(MADRS, DASS)
[122]	H; *L. rhamnosus*	+(depression, anxiety)
[123]	IBS with anx. or depr.; *B. longum*	+(HADS, QoL, brain activity)
[124]	H; *Lactobacilli*, *Bifidobacteria*	=(BDI, BAI)+(cognitive reactivity to sad mood)
[125]	MDD; *Lactobacilli*, *B. bifidum*	+(BDI, serum hs-CPR)
[126]	BD; *L. acidophilus*, *Bifidobacteria*	=(YMRS, HDRS)

Results mostly showed positive effects, but several studies could not find significant differences. “+” symbolizes a significantly positive health effect, while “=” indicates no significant difference. In brackets a selection of the investigated measures is given. Abbreviations: MDD, major depressive disorder; BD, bipolar disorder; D, participants with depressive symptoms; H, healthy participants; C, cross-sectional study; IBS, irritable bowel syndrome; BDI, Beck depression inventory; BAI, Beck anxiety inventory; DASS, depression anxiety stress scales; HADS, hospital anxiety and depression scale; PHQ-9, patient health questionnaire; HDRS, Hamilton depression rating scale; PSS, perceived stress scale; MADRS, Montgomery-Åsberg depression rating scale; BDNF, brain-derived neurotrophic factor; QoL, quality of life; hs-CPR, high-sensitivity C-reactive protein; YMRS, young mania rating scale.

### 3.8. Studies Involving Twins and Their Relatives

As genetics and environmental factors have a huge influence not only on the development of depression but also on the microbiome, three studies examined whether there is a correlation between the intestinal microbiota and depression in twins and relatives. Two studies investigated the difference of the microbiota in twins [53,54], and one study examined the difference between the microbiota of patients with newly diagnosed BD and their first-degree relatives [64].

The two twin studies investigated 128 monozygotic twins and one pair of monozygotic twins, respectively [53,54]. Vinberg et al. distinguished between affected twins (with a diagnosis of MDD or BD in remission), unaffected high-risk twins (with a co-twin history of depression), and low-risk twins (without any histories of depression in the family) [53]. They found a lower diversity and richness of the microbiota of affected twins, while high-risk twins showed the same pattern, but with the lower diversity only being a trend. Affected and high-risk twins also showed an absence of an OTU belonging to the family Christensenellaceae. However, no correlation of the microbiota with illness severity was found. Jiang et al. reported a less similar microbiota between the pair of discordant twins (one twin with a history of depression and one without) than the microbiota of two healthy spouses [54]. Moreover, the similarity of the microbiota reached its maximum after achieving full remission of the affected twin, with the level of Ruminococcaceae and *Faecalibacterium* increasing and *Enterobacter* decreasing during the responsive and remission periods. Several SCFA-producing genera, mainly belonging to the families Lachnospiraceae and Ruminococcaceae, were reduced in the active-BD state compared to the healthy spouses [54]. Further, an over-representation of LPS biosynthesis genes in the gut microbiota during the active depressive period was found, whereas in the remissive state these genes decreased, indicating a potential recovery of the microbiota during the responsive and remissive period. But with only one pair of monozygotic twins, these results must be taken with caution.

According to Coello et al., newly diagnosed BD subjects had a different microbiota, while the microbiota of unaffected first-degree relatives did not differ significantly from the microbiota of HC [64]. Especially the presence of *Flavonifractor* was associated with an increased odds ratio for having BD. After adjusting for smoking, this association attenuated, indicating an additional correlation of *Flavonifractor* with smoking, as well as with the female gender. It is hypothesized that the microbiota of BD patients is characterized by the different presence or absence of bacteria, especially of *Flavonifractor*, rather than the difference of bacterial abundance.

In summary, these studies support the hypothesis of a close connection between the intestinal microbiota and depression, and significant differences in the microbiota composition of depressed subjects were found. They even showed that a higher risk of developing depression is already associated with minor changes of the microbiota, and that with remission of depression, the intestinal bacteria change back towards a more “normal” composition. Comparing the reported bacterial alterations with our general findings, a beneficial effect of Christensenellaceae, Ruminococcaceae, and *Faecalibacterium*, and a negative effect of *Flavonifractor* show most experimental evidence.

## 4. Discussion

Our data show that the intestinal microbiota is closely linked with major depressive and bipolar disorder. The complexity of the microbiota makes it challenging to find clear causative associations, and a higher taxonomic resolution for determining the intestinal bacteria would be of importance for a more accurate analysis. Further studies with more participants are needed to verify specific bacterial alterations since the reviewed studies were mostly conducted with small sample sizes of up to 150 participants (with few exceptions). Study designs, inclusion criteria, analysis methods, and confounding factors varied widely, making a comparison difficult and may explain the contradicting results for certain bacteria. Therefore, a review of such heterogeneous studies is also associated with major limitations, and more standardized studies would facilitate a comparison.

Despite these limitations, this review demonstrated that certain bacteria consistently correlate with depression. The strongest and most consistent correlations are demonstrated in Table 8.

Additionally, phylum Bacteroidetes consistently positively correlated with depression in young individuals, whereas in middle-aged individuals a strong inverse correlation with depression was found, while phylum Firmicutes showed opposite correlations.

Noticeably, apart from phylum Bacteroidetes, Firmicutes, and Actinobacteria, the strongest correlations with depression were found on low taxonomic levels (particularly on genus level), underlining the importance of high taxonomic resolution to identify bacterial alterations in depressed subjects. Further studies specifically focusing on these altered bacteria and their interactions with the host could provide a better insight into the connection between depression and the human gut microbiota. SCFA-producing bacteria were mostly found to be reduced in depressed individuals, emphasizing the beneficial influence on their host. With a better understanding of the intestinal microbiota, new therapeutic strategies for the treatment of affective disorder could be found, which is crucial considering the high therapy resistance and relapse rates [5]. However, considering the complexity of the intestinal microbiota and the diversity of the bacterial changes found in this review, it is conceivable that bacterial clusters would show better correlations with depression. Consequently, studies with more participants are needed to identify depression-like bacterial clusters, as well as novel potential treatment approaches.

Changing the intestinal microbiota (for instance through specific diets, supplementations, probiotics, synbiotics, or fecal microbiota transplantation (FMT)) could potentially support the host’s health and mitigate depressive symptoms. While multiple clinical studies found probiotics and synbiotics to have a positive impact on mood and behavior, clinical FMT studies in depressed subjects remain scarce. FMT has repeatedly been shown to ameliorate depression [127]. After receiving fecal transplants of depressive patients, a depression-like behavior of germ-free mice was observed, compared to mice receiving fecal transplants of healthy individuals [63,128]. In a clinical study, patients with gastrointestinal complaints reported an improvement in depression scores after FMT, and in a case report, a treatment-resistant BD patient achieved full remission after FMT [56,129]. Further studies exploring the effect of probiotics, synbiotics and FMT with more individuals are required to strengthen these positive findings. In addition, since diet significantly influences the intestinal microtioba composition [112] and only few studies included dietary questionnaires, it is essential to adjust for dietary changes between depressed subjects and HC. Thereby bacterial alterations only due to different eating habits could be excluded in future studies.

Stevens et al. were able to differentiate depressed and non-depressed subjects using a machine learning approach [130]. Additional similar studies could offer the potential of finding specific bacterial clusters and changes in metabolic pathways associated with affective disorder. Moreover, it is suggested by these authors that this novel approach may be used as a reliable diagnostic tool to identify different depression phenotypes in the future, potentially even leading to personalized treatment of depression [130]. Even though reliably distinguishing between depressed and non-depressed individuals, it remains doubtful whether similar approaches would be of clinical importance as a diagnostic tool.

Another key question is how depressed subjects develop different abundances of certain intestinal bacteria. Does it depend on the diet, with depressed individuals showing different eating habits, do they have a special intestinal milieu that secondarily favors the colonization of certain bacteria, or are there other factors influencing the intestinal microbiota towards a depressive-like composition? Answering this question, which is the scope of another dedicated report, would help to prevent unfavorable microbiota changes and would provide further information about the bidirectional connection of the microbiota and depression.

While most of the studies only investigated MDD subjects, research with BD subjects is lagging, but an increasing number of studies including BD individuals in recent years shows a growing interest. In this review, we could not identify unequivocal differences between the microbiota abundance of MDD and BD subjects. Three studies juxtaposed MDD and BD individuals and found a distinct microbiota, but the results were controversial and inconsistent with the other studies including only MDD or BD subjects [46,50,74]. Therefore, a distinguishable microbiota is conceivable, but major differences could not be found.

In conclusion, strong correlations between the intestinal microbiota and affective disorder were found. Specifically investigating only MDD or BD individuals would decrease the heterogeneity of the disease manifestation, but other factors such as the analysis methods, subject heterogeneity, medication, nutrition, and lifestyle factors essentially confound the results. Additional standardized research is needed to elucidate the connection between the intestinal microbiota and depression and to further examine their interdependencies to eventually find novel therapeutic approaches and lower the rates of treatment-resistant affective disorder.

## Figures and Tables

**Table 3:** Different abundance of genus Bacteroides.

Source	Genus *Bacteroides*	
[42]	aMDD ↓	rMDD ↑
[45]	negative mood ↑	
[46]	BD ↑ (1 OTU)	
	MDD ↑/↓ (OTUs)	
[47]	anhedonia ↑	
	anxiety ↓	
[50]	MDD ↓, BD ↓	
[55]	anxiety ↑	
[57]	MDD ↑ (m)	
[60]	D ↑; IBS ↑; D + IBS ↑	
[66]	BD ↑	
[74]	MDD ↑	
[75]	BD ↑ (B-P group)	

Most studies reported an elevation correlating with affective disorder and depressive symptoms. “↓” and green symbolizes a significant reduction in diseased subjects or inverse correlation with more severe symptoms, while “↑” and orange shows a significant elevation or positive correlation with more severe symptoms, and grey symbolizes alterations in both directions or not evaluable. Brackets include additional information about the reported correlation (which bacteria showed a correlation or in which subgroup of subjects a correlation was found). Abbreviations: MDD, major depressive disorder; BD, bipolar disorder; D, depression in general; IBS, irritable bowel syndrome; B-P group, Bacteroides-Prevotella group; m, a correlation only in male subjects; a, active disease group; r, response group; OTU, operational taxonomic unit within genus Bacteroides.

**Table 4:** Different abundance of genus Roseburia.

Source	Genus *Roseburia*
[42]	aMDD ↑
[45]	↓ positive mood
[46]	BD ↓ (1 OTU)
[47]	anhedonia ↓
[48]	P ↓ remission (*R. inuliniforans*)
[52]	BD ↓ *
[55]	D ↓ (unclassified species)
[57]	MDD ↑ (f)
[60]	D ↓
[63]	MDD ↓ (OTUs)
[66]	BD ↓

Most studies reported a reduction correlating with affective disorder and negative mood. “↓” and green symbolizes a significant reduction in diseased subjects or inverse correlation with more severe symptoms, while “↑” and orange shows a significant elevation or positive correlation with more severe symptoms, and grey symbolizes only a trend. Brackets include additional information about the reported correlation (which bacteria showed a correlation or in which subgroup of patients a correlation was found). Abbreviations: MDD, major depressive disorder; BD, bipolar disorder; D, depression in general; f, a correlation only in female subjects; a, active disorder; OTU, operational taxonomic unit within genus Roseburia; *, only negatively correlating with symptom severity, but not significantly correlating with BD compared to healthy controls.

**Table 5:** Different abundance of family Ruminococcaceae and two of its members, genus Ruminococcus and Faecalibacterium.

Source	Family Ruminococcaceae	Genus *Ruminococcus*	Genus *Faecalibacterium*
[42]	MDD ↓	aMDD ↓	MDD ↓
[44]	MDD ↓	MDD ↓ (*Ruminococcus 1*)	MDD ↓
[45]			negative mood ↑
[46]	MDD ↓ (OTUs)		
	BD ↑/↓ (OTUs)		
[47]	anhedonia ↓		
[48]	P ↓ remission	P ↑ (*Ruminococcus 1*)	P ↓ remission (*F. prausnitzii*)
[49]	MDD ↑/↓ (OTUs)		
[51]		MDD ↑	
[52]	BD ↓		BD ↓
[54]	BD ↓		BD ↓
[55]			D ↑ (unclassified species)
[57]	MDD ↑/↓ (OTUs)		MDD ↑ (f)
[58]	MDD ↓		
[60]		D ↓	
[63]	MDD ↑ (OTUs)		MDD ↓ (OTUs)
[66]		BD ↓	BD ↓
[67]	MDD ↑		MDD ↓
[75]			BD ↑ (*F. prausnitzii*)
[77]			lower QoL ↓
[99]		DASS ↑ (*R. gnavus*)	
[100]	BD ↓ (1 OTU)		BD ↓

Most studies reported an elevation of these bacteria correlating with better health. “↓” and green symbolizes a significant reduction in diseased subjects or inverse correlation with more severe symptoms, while “↑” and orange shows a significant elevation or positive correlation with more severe symptoms, and grey symbolizes alterations in both directions. Brackets include additional information about the reported correlation (which bacteria showed a correlation or in which subgroup of patients a correlation was found). Empty cells symbolize that no significant results were reported. Abbreviations: MDD, major depressive disorder; BD, bipolar disorder; D, depression in general; P, psychiatric subjects; DASS, depression anxiety stress scales; QoL, quality of life; f, a correlation only in female subjects; a, active disorder; OTU, operational taxonomic unit.

**Table 6:** Different abundances of family Bifidobacteriaceae and genus Bifidobacterium.

Source	Family Bifidobacteriaceae		Genus *Bifidobacterium*
[43]			MDD ↑
[45]			negative mood ↑
[46]	MDD ↑	BD =	
[47]	anhedonia ↓		anhedonia ↓
[50]			MDD ↑, BD ↑
[51]	MDD ↑		MDD ↑
[56]			HDRS ↑ (*B. longum*)
[57]			MDD ↑ (f)
[67]	MDD ↑		
[79]			BD =
[113]			MDD ↓

Results tended to show a negative effect of an elevation of these bacteria. “↓” and green symbolizes a significant reduction in diseased subjects or inverse correlation with more severe symptoms, while “↑” and orange shows a significant elevation or positive correlation with more severe symptoms, and “=” and grey symbolizes no correlation. Brackets include additional information about the reported correlation (which bacteria showed a correlation or in which subgroup of patients a correlation was found). Empty cells symbolize that no significant results were reported. Abbreviations: MDD, major depressive disorder; BD, bipolar disorder; HDRS, Hamilton depression rating scale; f, a correlation only in female subjects.

**Table 8:** Bacteria (with taxonomic level) that correlated most with depression.

More Abundant in Depressive Subjects	Less Abundant in Depressive Subjects
Actinobacteria (phylum)	Christensenellaceae and *Christensenella* (family and genus)
*Alistipes* (genus)	*Coprococcus* (genus)
*Bacteroides* (genus)	*Eubacterium* and *E. rectale* (genus and species)
Bifidobacteriaceae and *Bifidobacterium* (family and genus)	*Faecalibacterium* and *F. prausnitzii* (genus and species)
*Flavonifractor* (genus)	*Roseburia* (genus)
*Parabacteroides* (genus)	Ruminococcaceae (family)
*Streptococcus* (genus)	Sutterellaceae and *Sutterella* (family and genus)

## Abbreviations

ACTH	Adrenocorticotrophin
BAI	Beck anxiety inventory
BD	Bipolar disorder
BDI	Beck depression inventory
BDNF	Brain-derived neurotrophic factor
BMI	Body mass index
COVID-19	Coronavirus disease 2019
CRP	C-reactive protein
DASS	Depression anxiety stress scales
FMT	Fecal microbiota transplantation
FGID	Functional gastrointestinal disorders
GABA	Gamma-aminobutyric acid
HADS	Hospital anxiety and depression scale
HC	Healthy controls
HDRS	Hamilton depression rating scale
HPA axis	Hypothalamic-pituitary-adrenal axis
IBD	Inflammatory bowel disease
IBS	Irritable bowel syndrome
IgM	Immune globulin M
LPS	Lipopolysaccarides
MADRS	Montgomery-Åsberg depression rating scale
MDD	Major depressive disorder
OTU	Operational taxonomic unit
PANDAS	Pediatric autoimmune neuropsychiatric disorders associated with streptococcal infections
PHQ-9	Patient health questionnaire
PLS-DA	Partial least squares discriminant analysis
PSS	Perceived stress scale
PUFA	Polyunsaturated fatty acids
QoL	Quality of life
SCFA	Short-chain fatty acids
spp.	Species
YMRS	Young mania rating scale
